# Review of "Mathematical Models In Biology" 6th Edition, by Leah Eldestein-Keshet

**DOI:** 10.1186/1475-925X-4-35

**Published:** 2005-06-07

**Authors:** Giberto Chirico

**Affiliations:** 1 Department of Physics, University of Milano Bicocca, Piazza della Scienza 3, Milano, 20126 Italy

This book by Leah Edelstein-Keshet, "Mathematical Models in Biology," is a discovery that delighted me at once. It is simple to read and well organized, with basics of mathematics given in chapters separated from applications and examples. At first glance it might be taken more as a book for researchers already involved in the fields of biology, biophysics, and physical medicine rather than a text book for graduate students. To be sure, many of the biologists who are slowly discovering that mathematics is essential to understand modern biology and medicine, will find this book to be a guided tour to many of the mathematical fields connected to the life sciences. However, also students in the mathematical and physical sciences will find in this book a detailed guide to the field of mathematical modeling biological processes.

Although some of the newest fields in mathematical modeling of biological systems are only cited in this text, the way the basic ideas in modeling are presented is an essential step for future studies. This major goal of the book is reached by always giving the basic tools of mathematics and calculus in short separate chapters or in boxes in the text. Few mathematical concepts are needed for a reader who is new to this field. Moreover, the author expends much effort in presenting and explaining the methods of mathematical modeling of biological problems. Many examples are discussed in the text, and numerous others are given as guided exercises at the end of each chapter.

These features would actually allow the book to be used as an excellent text book in courses for biophysics or biology curriculums. But more than this, the text offers deep insights into higher mathematical concepts and methods that are extremely useful for active researchers.

In summary, "Mathematical Models in Biology" by Leah Edelstein-Keshet is an essential step for students and researchers active in a wide variety of fields; from cell and molecular biophysics to classical biology.

